# U-shaped association between triglyceride-glucose index and all-cause mortality among critically ill pediatrics: a population-based retrospective cohort study

**DOI:** 10.1186/s12933-024-02310-2

**Published:** 2024-06-26

**Authors:** Qi Gao, Fan Luo, Hongxue Yu, Yuxin Lin, Ruqi Xu, Pingping Li, Yuping Zhang, Jiao Liu, Licong Su, Yanqin Li

**Affiliations:** 1 State Key Laboratory of Organ Failure Research, Division of Nephrology, National Clinical Research Center for Kidney Disease, Nanfang Hospital, Southern Medical University, 1838 N Guangzhou Ave, Guangzhou, 510515 China; 2 Division of Nephrology, People’s Hospital of Yangjiang Affiliated to Guangdong Medical University, Yangjiang, China

**Keywords:** TyG index, All-cause mortality, Pediatrics, Critically ill, PIC database

## Abstract

**Background:**

Previous studies have shown that an elevated triglyceride-glucose (TyG) index was associated with all-cause mortality in both general adult individuals and critically ill adult patients. However, the relationship between the TyG index and clinical prognosis in pediatric patients admitted to the intensive care unit (ICU) remains unknown. We aimed to investigate the association of the TyG index with in-hospital all-cause mortality in critically ill pediatric patients.

**Methods:**

A total of 5706 patients in the Pediatric Intensive Care database were enrolled in this study. The primary outcome was 30-day in-hospital all-cause mortality, and secondary outcome was 30-day in-ICU all-cause mortality. The restricted cubic spline (RCS) curves and two-piecewise multivariate Cox hazard regression models were performed to explore the relationship between the TyG index and outcomes.

**Results:**

The median age of the study population was 20.5 [interquartile range (IQR): 4.8, 63.0] months, and 3269 (57.3%) of the patients were male. The mean TyG index level was 8.6 ± 0.7. A total of 244 (4.3%) patients died within 30 days of hospitalization during a median follow-up of 11 [7, 18] days, and 236 (4.1%) patients died in ICU within 30 days of hospitalization during a median follow-up of 6 [3, 11] days. The RCS curves indicated a U-shape association between the TyG index and 30-day in-hospital and in-ICU all-cause mortality (both *P* values for non-linear < 0.001). The risk of 30-day in-hospital all-cause mortality was negatively correlated with the TyG index until it bottoms out at 8.6 (adjusted hazard ratio [HR], 0.72, 95% confidence interval [CI] 0.55–0.93). However, when the TyG index was higher than 8.6, the risk of primary outcome increased significantly (adjusted HR, 1.51, 95% CI 1.16–1.96]). For 30-day in-ICU all-cause mortality, we also found a similar relationship (TyG < 8.6: adjusted HR, 0.75, 95% CI 0.57–0.98; TyG ≥ 8.6: adjusted HR, 1.42, 95% CI 1.08–1.85). Those results were consistent in subgroups and various sensitivity analysis.

**Conclusions:**

Our study showed that the association between the TyG index and 30-day in-hospital and in-ICU all-cause mortality was nonlinear U-shaped, with a cutoff point at the TyG index of 8.6 in critically ill pediatric patients. Our findings suggest that the TyG index may be a novel and important factor for the short-term clinical prognosis in pediatric patients.

**Supplementary Information:**

The online version contains supplementary material available at 10.1186/s12933-024-02310-2.

## Introduction

Patients in intensive care unit (ICU) usually have a markedly high mortality rates, especially in pediatrics. Previous studies have developed scores such as the pediatric risk of mortality (PRISM) and the pediatric index of mortality (PIM) to identify mortality risk [1, 2]. However, these scores are limited in predicting outcomes due to the large number of clinical variables required. Therefore, it is essential to investigate more accessible and effective risk factors for mortality in ICU patients. The triglyceride-glucose (TyG) index, calculated based on fasting blood glucose (FBG) and triglyceride (TG) levels, has been proposed as a reliable surrogate marker for insulin resistance (IR) [3–6]. Published studies have shown that elevated TyG index was associated with diabetes, cardiovascular and cerebrovascular diseases, and all-cause mortality, and these studies mainly focused on adult individuals [7–15]. However, the prognostic value of TyG index in pediatric populations remains uncertain. A systematic review comprising eight cross-sectional studies has suggested that TyG index may serve as a predictive factor for IR and cardiovascular metabolic risks in school-aged and adolescent children [16]. Notably, the study primarily focused on relatively healthy children aged 4–20 years, and the relationship between TyG index and clinical prognosis in younger and critically ill children warrants further investigation.

Therefore, based on the Pediatric Intensive Care (PIC) database, our study aimed to explore the association between the TyG index and in-hospital all-cause mortality in critically ill pediatric patients.

## Methods

### Study design, data source, and population

The study population was derived from the PIC database, which is an open, single-center, retrospective electronic medical record database developed by the Children’s Hospital, Zhejiang University School of Medicine (ZUCH). Detailed information about this database can be found on the official website (http://pic.nbscn.org) as well as in previous research studies [17–21]. In brief, the ZUCH, with more than 1900 beds, is the largest comprehensive pediatric medical center in Zhejiang Province, and it has 119 critical care beds in 5 ICUs, including general ICU (GICU), pediatric ICU (PICU), surgical ICU (SICU), cardiac ICU (CICU), and neonatal ICU (NICU). The PIC database contains clinical data from 12,881 pediatric patients admitted to any ICU in the ZUCH from 2010 to 2018. The clinical data included laboratory tests, prescriptions, diagnoses, physical examinations, surgical procedures, and admission and discharge date. The project has been approved by the Ethics Committee of the ZUCH, and the requirement of individual patients informed consent was waived because the retrospective nature of the study and all personalized information has been de-identified.

**Fig. 1:**
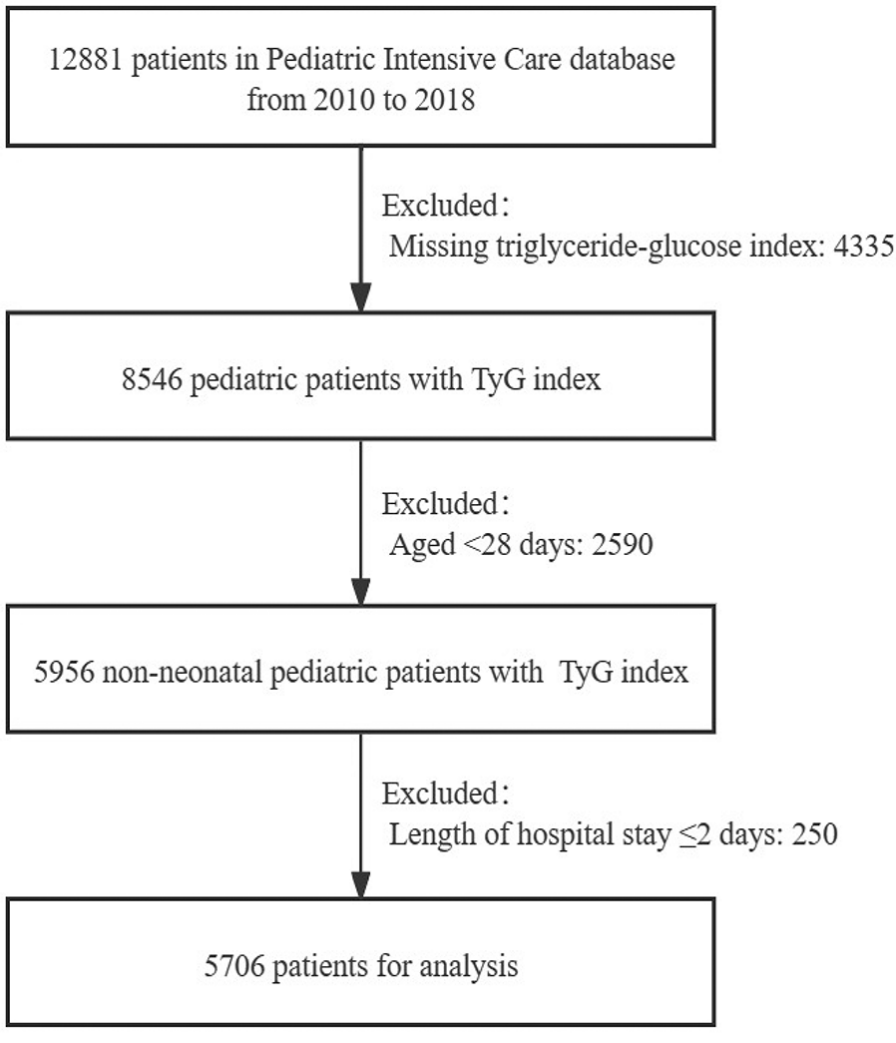
Flowchart of study population selection

This study included all 12,881 patients from the PIC database. The exclusion criteria were as follows: (a) missing TyG index (n = 4335); (b) age ≤ 28 days (n = 2590); (c) length of hospital stay (LOS hospital) ≤ 2 days (n = 250). Finally, a total of 5706 pediatric patients were included in the final analysis. The flowchart of the study population is shown in Fig. 1.

### Clinical variables

The study variables included demographic information, LOS hospital, length of ICU stay (LOS ICU), ICU type, medication treatment, clinical diagnoses, surgical procedures, and laboratory tests. The exposure window of all laboratory tests, medication treatments, and surgical procedures was defined within 48 h after admission. If the laboratory tests were measured multiple times after admission, data from the first time were used. Clinical diagnoses were determined based on International Classification of Diseases 10 (ICD-10) codes.

### Exposure, outcomes, and follow-up

The TyG index was calculated using the equation: ln (TG (mg/dL) × FBG (mg/dL)/2) [22]. The primary outcome of this study was 30-day in-hospital all-cause mortality after admission, and the secondary outcome was 30-day in-ICU all-cause mortality, namely, death occurring in ICU after admission. For the primary outcome, patients were followed up until death, the 30th days after admission, or discharged from the hospital, whichever came first. For secondary outcome, patients were followed up until death, the 30th days after admission, or discharged from ICU, whichever came first.

### Handling of missing values

In the dataset, there were missing values in blood urea nitrogen (BUN, n = 1), cystatin C (n = 1), serum creatinine (SCr, n = 3), hemoglobin (n = 10), white blood cell count (WBC, n = 61), platelets (n = 61), high-sensitivity C-reactive protein (hs-CRP, n = 67), neutrophil percentage (n = 73), and lymphocyte percentage (n = 116). We performed imputation of missing values using the random forest method in the R package RandomForest [23].

### Statistical analysis

Continuous variables were presented as mean ± standard deviation (SD) or median [interquartile range (IQR)] and compared between groups using analysis of variance (ANOVA) and the Kruskal–Wallis test. Categorical variables were presented as frequencies (percentages) and compared between groups using the chi-square test.

We explored the association between the TyG index and mortality using univariate and multivariate Cox proportional hazards models, and hazard ratios (HRs) were expressed with their 95% confidence intervals (95% CIs). The Model 1 adjusted for age and sex, ICU types (including GICU, PICU, SICU, CICU, and NICU), surgical procedures (including cardiac surgery, neurosurgery, gastrointestinal surgery, respiratory system surgery, and other surgeries), congenital valvular heart disease, congenital heart disease, malignancy, pneumonia, sepsis, shock, use of vasopressors, WBC, lymphocyte percentage, neutrophil percentage, and hs-CRP. Model 2 further adjusted for other biochemical markers including serum albumin, total cholesterol, hemoglobin, platelets, BUN, cystatin C, and SCr. We used restricted cubic spline (RCS) Cox regression (adjusted for variables in Model 2, with 3 knots at the 10th, 50th, and 90th percentiles) to explore the dose–response relationship between the TyG index and the 30-day in-hospital and in-ICU all-cause mortality. The median of the TyG index was adopted as our reference, a widely accepted approach due to the absence of a recognized standard for the TyG index [24, 25]. Finally, based on the results of RCS analysis, we determined the optimal threshold and conducted threshold analysis using two-piecewise Cox regression to explore the relationship between the TyG index and the endpoints.

#### Subgroup analyses

To explore potential effect modifiers, we performed several subgroup analyses. Patients were stratified by age (1 month to 1 year, 1 year to 5 year, and ≥ 5 years), sex (male and female), ICU types (GICU, PICU, and other ICU), surgical procedures (yes or no), congenital heart disease (yes or no), sepsis (yes or no), and use of vasopressors (yes or no).

#### Sensitivity analyses

The robustness of results was further validated by several sensitivity analyses. First, we performed sensitivity analyses 1, 2, and 3 to minimize reverse causation between the TyG index and short-term mortality. Then, we performed sensitivity analyses 4 to explore whether missing values had an impact on the primary results. Sensitivity analysis 1: we excluded patients with severe conditions, including those with sepsis, shock, pneumonia, and malignancy. Sensitivity analysis 2: based on sensitivity analysis 1, we further excluded patients diagnosed with acute kidney injury (AKI) during hospital stay. AKI was identified using Kidney Disease Improving Global Outcomes (KDIGO) SCr criteria, defined as SCr during the follow-up ≥ 1.5 fold baseline SCr or need for dialysis [26, 27]. Sensitivity analysis 3: we excluded patients with LOS hospital less than 3 days. Sensitivity analysis 4: we reanalyzed using the dataset before imputation.

#### Additional analyses

The E-value analysis has been widely used in observational studies to assess the impact of unmeasured or uncontrolled confounding on study outcomes [28, 29]. It evaluated the minimum strength of association that an unmeasured confounder would need to have with both the exposure and the outcome to nullify the observed exposure-outcome association. Briefly, if the relative risk between unmeasured confounders, mortality and the TyG index over the computed E-value, residual confounding may be a plausible explanation for the detected association.

All analyses were conducted using R software (version 4.1.2, http://www.r-project.org/). A two-tailed P value less than 0.05 was considered statistically significant.

## Results

### Study population and baseline characteristic

A total of 5706 critically ill pediatric patients were included in the final analysis. Baseline characteristics of the study population stratified by TyG levels are presented in Table 1. Of the 5706 patients, 3269 (57.3%) were males, with a median age of 20.5 [4.8, 63.0] months. The mean TyG index was 8.6 ± 0.7, and the median time from hospital admission to ICU admission was 1 day. Compared to patients with TyG < 8.6, those with higher TyG levels (TyG ≥ 8.6) tended to be younger, had a lower proportion of males, a higher proportion of undergoing cardiac surgeries, congenital heart disease, and use of vasopressors. LOS hospital was similar between the two groups. Baseline characteristics of the study population stratified by TyG levels and outcome are shown in Supplementary Table 1.

**Table 1:** Study population characteristics stratified by TyG index level

Characteristics	TyG index			
	Overall (N = 5706)	$\ge$ 8.6 (N = 2926)	< 8.6 (N = 2780)	P value
TyG index	8.6 ± 0.7	9.1 ± 0.5	8.1 ± 0.5	< 0.001
Male, %	3269 (57.3)	1611 (55.1)	1658 (59.6)	0.001
Age, months	20.5 [4.8, 63.0]	17.5 [5.0, 56.6]	24.0 [4.5, 69.5]	0.002
Age category (%)				< 0.001
1 month-1 year	2239 (39.2)	1225 (41.9)	1014 (36.5)	
1 year-5 years	1980 (34.7)	1021 (34.9)	959 (34.5)	
$\ge$ 5 years	1487 (26.1)	680 (23.2)	807 (29.0)	
ICU types (%)				< 0.001
GICU	1357 (23.8)	730 (24.9)	627 (22.6)	
CICU	883 (15.5)	550 (18.8)	333 (12.0)	
NICU	335 (5.9)	102 (3.5)	233 (8.4)	
PICU	1450 (25.4)	684 (23.4)	766 (27.6)	
SICU	1681 (29.5)	860 (29.4)	821 (29.5)	
Laboratory				
Albumin, g/L	41.5 [36.6, 45.1]	41.6 [36.8, 45.2]	41.4 [36.5, 44.9]	0.306
BUN, mmol/L	3.72 [2.61, 4.96]	3.73 [2.50, 5.09]	3.71 [2.73, 4.86]	0.740
Cystatin-C, mg/dL	0.88 [0.71, 1.17]	0.92 [0.75, 1.22]	0.83 [0.68, 1.11]	< 0.001
SCr, mmol/L	43.9 [37.0, 52.0]	43.0 [37.0, 52.0]	44.0 [36.0, 52.0]	0.614
Total cholesterol, mmol/L	3.61 [2.90, 4.32]	3.77 [3.08, 4.46]	3.42 [2.73, 4.12]	< 0.001
Hemoglobin, g/L	114.0 [99.0, 125.0]	113.0 [98.0, 125.0]	115.0 [100.0, 126.0]	< 0.001
hs-CRP, mg/L	3.0 [1.0, 10.0]	3.0 [1.0, 9.0]	3.0 [1.0, 10.0]	0.046
Lymphocyte, %	42.0 [23.8, 58.4]	46.4 [27.3, 61.9]	38.1 [20.8, 53.8]	< 0.001
Neutrophil, %	47.5 [30.1, 67.9]	42.5 [26.9, 63.1]	52.0 [34.4, 72.6]	< 0.001
Platelet, 10^9^/L	312.0 [227.0, 398.0]	318.0 [227.0, 410.0]	306.0 [228.0, 386.0]	0.001
WBC,10^9^/L	9.0 [6.7, 12.3]	9.2 [6.8, 12.5]	8.9 [6.6, 12.1]	0.027
Surgical procedures (%)				
Cardiac	624 (10.9)	403 (13.8)	221 (7.9)	< 0.001
Gastrointestinal	214 (3.8)	92 (3.1)	122 (4.4)	0.016
Neurosurgical	521 (9.1)	269 (9.2)	252 (9.1)	0.902
Respiratory	99 (1.7)	51 (1.7)	48 (1.7)	1.000
Other	125 (2.2)	79 (2.7)	46 (1.7)	0.009
Comorbidities (%)				
Congenital valvular heart disease	67 (1.2)	43 (1.5)	24 (0.9)	0.045
Congenital heart disease	763 (13.4)	475 (16.2)	288 (10.4)	< 0.001
Pneumonia	628 (11.0)	299 (10.2)	329 (11.8)	0.057
Malignant tumors	283 (5.0)	203 (6.9)	80 (2.9)	< 0.001
Sepsis	177 (3.1)	97 (3.3)	80 (2.9)	0.381
Shock	29 (0.5)	15 (0.5)	14 (0.5)	1.000
Vasopressors use, %	1103 (19.3)	624 (21.3)	479 (17.2)	< 0.001
LOS hospital, days	11.0 [7.0, 18.0]	11.0 [7.0, 18.0]	11.0 [7.0, 18.0]	0.445
LOS ICU, days	2.8 [0.9, 7.7]	2.5 [0.9, 7.0]	2.9 [0.9, 8.2]	0.012
Days from hospital admission to ICU admission	1.0 [0.0, 2.0]	1.0 [0.0, 2.0]	1.0 [0.0, 3.0]	< 0.001

*TyG* triglyceride-glucose, *ICU* intensive care unit, *GICU* general intensive care unit, *PICU* pediatric intensive care unit, *SICU* surgical intensive care unit, *CICU* cardiac intensive care unit, *NICU* neonatal intensive care unit, *BUN* blood urea nitrogen, *SCr* serum creatine, *WBC* white blood cell count, *hs-CRP* high sensitivity C reactive protein, *LOS* length of stay

### Association between Tyg index and 30-day mortality

**Fig. 2:**
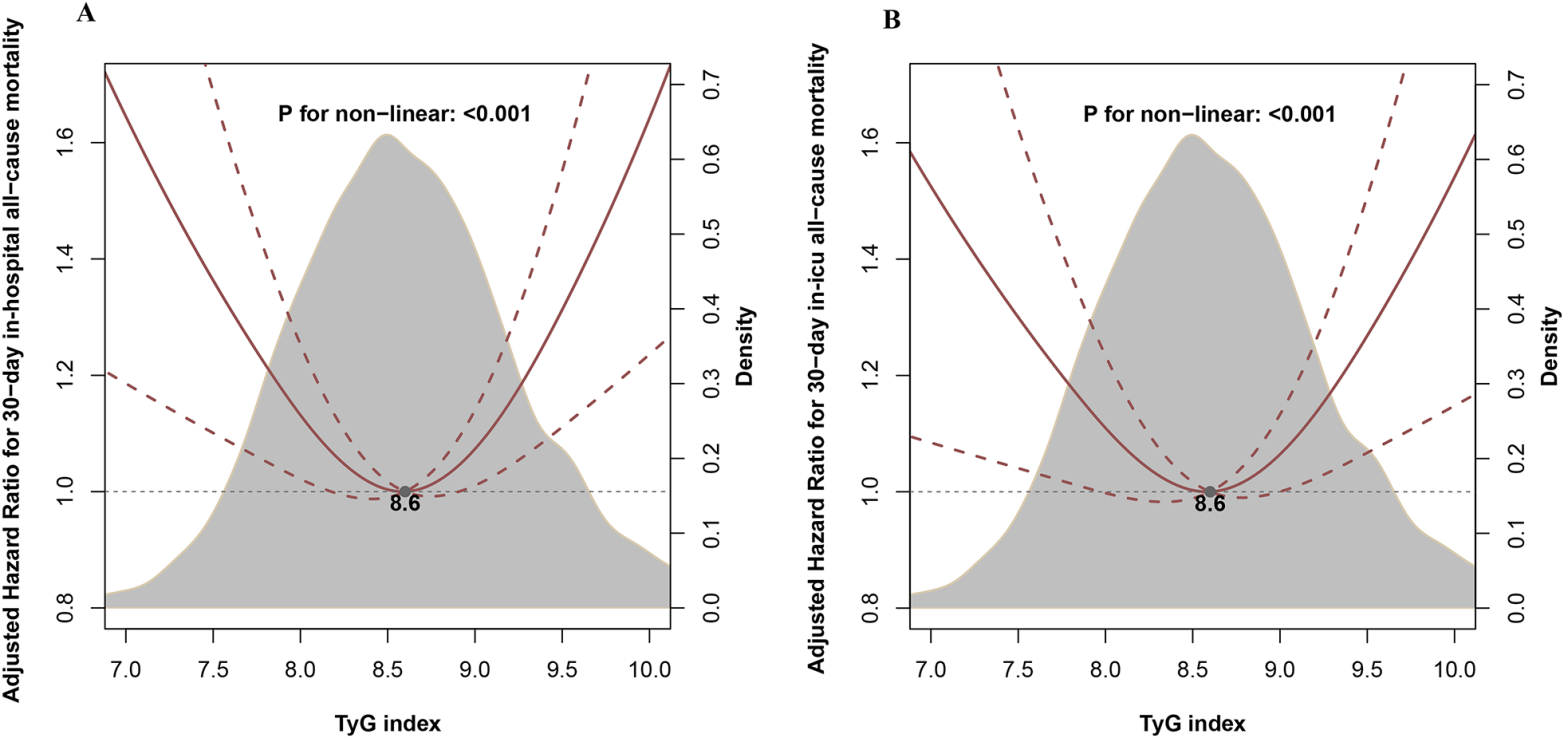
Relationship between the TyG index and the risk of 30-day in-hospital all-cause mortality and 30-day in-ICU all-cause mortality based on restricted cubic spline curves. *TyG* triglyceride-glucose, *ICU* intensive care unit. **A** 30-day in-hospital all-cause mortality, **B** 30-day in-ICU all-cause mortality. The model was adjusted for age, sex, ICU types, surgical procedure, congenital valvular heart disease, congenital heart disease, malignant tumors, pneumonia, sepsis, shock, use of vasopressors, white blood cell count, lymphocyte percentage, neutrophil percentage, high sensitivity C reactive protein, serum albumin, total cholesterol, hemoglobin, platelets, blood urea nitrogen, cystatin C, and serum creatinine

Overall, during a median follow-up period of 11 days (IQR: 7 to 18 days), 4.3% (244 out of 5706) of patients occurred 30-day in-hospital all-cause mortality, and for secondary outcome, during a median follow-up period of 6 days (IQR: 3 to 11 days), 4.1% (236 out of 5706) of patients occurred 30-day in-ICU all-cause mortality. After adjusting for age, sex, ICU and surgical types, vasopressors, laboratory tests, and comorbidities, RCS curves showed a U-shaped association between the TyG index and outcomes, with a threshold of 8.6 (both *P* values for non-linear < 0.001, Fig. 2). This indicates that the TyG index demonstrates distinct associations with outcomes before and after the cutoff point. Specifically, the multivariable two-piecewise Cox model showed that a per unit increase in the TyG index was associated with a 28% decrease in the risk of 30-day in-hospital all-cause mortality (adjusted HR, 0.72, 95% CI 0.55–0.93) in patients with a TyG index < 8.6, and was associated with a 51% increase in the risk of 30-day in-hospital all-cause mortality (adjusted HR, 1.51, 95% CI 1.16–1.96) in patients with a TyG index ≥ 8.6. A similar trend was observed in secondary outcome, the risk of 30-day in-ICU all-cause mortality was lower with an increase in the TyG index (adjusted HR, 0.75, 95% CI 0.57–0.98) in patients with a TyG < 8.6, and higher with an increase in the TyG index (adjusted HR, 1.42, 95% CI 1.08–1.85) in patients with a TyG index ≥ 8.6 (Table 2).

**Table 2:** Association between TyG index and outcomes in critical ill pediatric patients

TyG index (per unit increase)	Total, N	No. of events (incident rate, %)	Crude model		Model 1		Model 2	
			HR [95% CI]	P value	HR [95% CI]	P value	HR [95% CI]	P value
Primary outcome: 30-day in-hospital all-cause mortality								
< 8.6	2780	108 (3.9)	0.69 [0.55, 0.85]	0.001	0.72 [0.56, 0.94]	0.014	0.72 [0.55, 0.93]	0.014
$\ge$ 8.6	2926	136 (4.7)	2.03 [1.61, 2.56]	< 0.001	1.80 [1.42, 2.30]	< 0.001	1.51 [1.16, 1.96]	0.002
Secondary outcome: 30-day in-ICU all-cause mortality								
< 8.6	2780	105 (3.8)	0.69 [0.53, 0.89]	0.004	0.74 [0.57, 0.98]	0.033	0.75 [0.57, 0.98]	0.035
$\ge$ 8.6	2926	131 (4.5)	1.89 [1.50, 2.38]	< 0.001	1.67 [1.30, 2.14]	< 0.001	1.42 [1.08, 1.85]	0.011

Model 1 adjusted for age, sex, ICU types, surgical procedure, congenital valvular heart disease, congenital heart disease, malignant tumors, pneumonia, sepsis, shock, use of vasopressors, white blood cell count, lymphocyte percentage, neutrophil percentage, and high sensitivity C reactive protein

Model 2 further adjusted for serum albumin, total cholesterol, hemoglobin, platelets, blood urea nitrogen, cystatin C, and serum creatinine

*TyG* triglyceride-glucose, *ICU* intensive care unit, *HR* hazard ratio, *CI* confidence interval

### Subgroup analyses

**Fig. 3:**
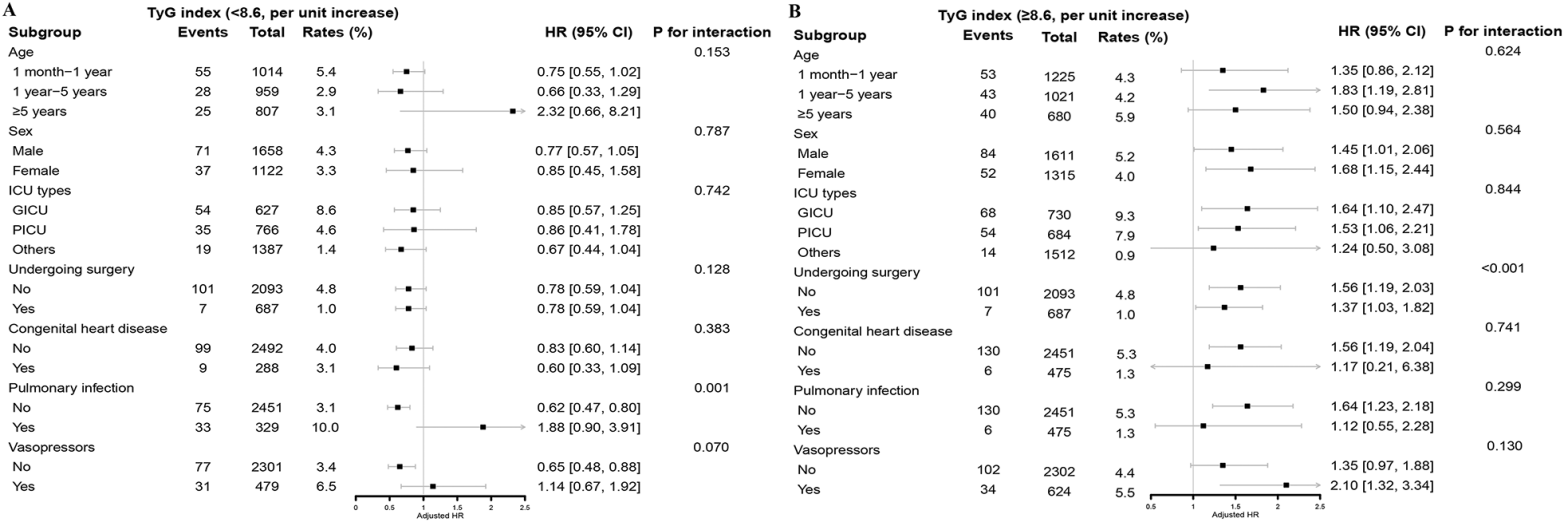
Stratified analyses by potential modifiers of the association between TyG index and 30-day in-hospital all-cause mortality. *TyG* triglyceride-glucose, *ICU* intensive care unit, *HR* hazard ratio, *CI* confidence interval. **A** TyG index < 8.6, **B** TyG index ≥ 8.6. The model was adjusted for age, sex, ICU types, surgical procedure, congenital valvular heart disease, congenital heart disease, malignant tumors, pneumonia, sepsis, shock, use of vasopressors, white blood cell count, lymphocyte percentage, neutrophil percentage, high sensitivity C reactive protein, serum albumin, total cholesterol, hemoglobin, platelets, blood urea nitrogen, cystatin C, and serum creatinine

The relationship between the TyG index and 30-day in-hospital all-cause mortality was further explored in different subgroups. The association was consistent across various subgroups, including age, sex, ICU types, congenital heart disease, sepsis, and use of vasopressors. Moreover, we found that pulmonary infection was a modified factor in patients with a TyG index < 8.6 (without pulmonary infection: adjusted HR, 0.62, 95% CI 0.47–0.80; with pulmonary infection: adjusted HR, 1.88, 95% CI 0.90–3.91; *P* for interaction = 0.001, Fig. 3). Furthermore, surgery was found had a modifying effect in patients with a TyG index ≥ 8.6 (without undergoing surgery: adjusted HR, 1.56, 95% CI 1.19–2.03; undergoing surgery: adjusted HR, 1.37, 95% CI 1.03–1.82, *P* for interaction < 0.001, Fig. 3).

### Sensitivity analyses

The results remained consistent with the primary results in several sensitivity analyses. Excluding patients at high risk of short-term mortality did not substantially alter the effect of TyG index on 30-day in-hospital all-cause mortality (sensitivity analysis 1: TyG index < 8.6, adjusted HR, 0.61, 95% CI 0.47–0.78; TyG index ≥ 8.6, adjusted HR, 1.69, 95% CI 1.24–2.30; sensitivity analysis 2: TyG index < 8.6, adjusted HR, 0.60, 95% CI 0.45–0.80; TyG index ≥ 8.6, adjusted HR, 1.84, 95% CI 1.29–2.63, Supplementary Table 2). In addition, after we modified the follow-up from ≤ 2 days to ≤ 3 days, the association was slightly attenuated (sensitivity analysis 3: TyG index < 8.6, adjusted HR, 0.73, 95% CI 0.55–0.98; TyG index ≥ 8.6, adjusted HR, 1.45, 95% CI 1.08–1.95, Supplementary Table 2). Finally, when we utilized the dataset before imputation, the results were similar to the primary analysis (sensitivity analysis 4: TyG index < 8.6, adjusted HR, 0.72, 95% CI 0.55–0.93; TyG index ≥ 8.6, adjusted HR, 1.47, 95% CI 1.12–1.93, Supplementary Table 2).

### Additional analyses

The E-values for TyG index < 8.6 and ≥ 8.6 with respect to in-hospital 30-day all-cause mortality were calculated as 2.12 and 2.19, respectively. In comparison, the HRs for using vasopressors and having congenital heart disease and the outcome were 2.12 and 2.30 in the model 2, respectively. Using the E-values, we found that an unmeasured confounder would need to be associated with both TyG index and mortality by a risk ratio of roughly 2.12 (at least) to explain away the association, which we believe is unlikely.

## Discussion

This large, single-center cohort study included 5706 critically ill pediatric patients from the PIC database. After adjusting for various confounding factors, we found a U-shaped nonlinear association between the TyG index and 30-day in-hospital as well as in-ICU all-cause mortality, with an inflection point at TyG index of 8.6. This association remained consistent across various subgroups and in several sensitivity analyses. To our knowledge, this is the first study to explore the relationship between the TyG index and short-term mortality in critically ill pediatric patients. Our study showed that the TyG index was independently associated with in-hospital mortality, providing some new insights into preventing mortality among critically ill pediatrics.

Current researches on the association between the TyG index and critically ill adult patients mainly focused on those with cardiovascular and cerebrovascular diseases [11, 13–15]. Zhang et al. used the eICU database and found that in critically ill patients with ischemic stroke, each unit increase in the the TyG index was associated with a 45% higher risk of in-hospital mortality and a 74% increased risk of in-ICU mortality, and this association was not observed in those with hemorrhagic stroke [11]. Subsequently, Cai et al. further validated these results in critically ill patients with ischemic stroke using the Medical Information Mart for Intensive Care (MIMIC) database [13]. Conversely, Chen et al. observed a significant association between the TyG index and in-hospital mortality risk in patients with hemorrhagic stroke (TyG index, quantile 4 VS. quantile 1, adjusted Odds Ratio, 8.9, 95% CI 3.5–24.2) [14]. Only two studies have explored the relationship between the TyG index and in-hospital outcomes in all critically ill patients, both of which enrolled adult populations. Their findings consistently indicated a positive correlation between the TyG index and in-hospital and in-ICU mortality [12, 30]. To date, no studies have investigated the association between the TyG index and in-hospital mortality or in-ICU mortality in critically ill pediatric patients, and our study filled this knowledge gap.

Consistent with the aforementioned studies involving critically ill adult patients, we found that the TyG index was an independent risk factor for in-hospital and in-ICU mortality in pediatric patients. Furthermore, our study revealed a nonlinear U-shaped relationship between the TyG index and mortality, which differed from the typically reported a linear or nonlinear J-shaped relationship in previous studies [11–15, 31]. This discrepancy might be attributed to different distribution of the TyG index among study populations. In our study, the TyG index was 8.6 [8.2, 9.0] and 8.6 ± 0.7, whereas previous studies generally reported higher TyG index levels than ours. These studies had fewer patients with lower levels of TyG index, which may fail to fully reveal the relationship between this subset of adult patients and mortality. On the other hand, a low FBG level could lead to a low TyG index, and hypoglycemia in ICU patients was associated with an increased risk of mortality [32, 33], which might explain the negative correlation observed between the TyG index and mortality risk when the TyG index was relatively low (TyG index < 8.6).

When the TyG index was < 8.6 and in the subgroup of patients with pulmonary infection, we found a positive association trend between the TyG index and in-hospital mortality (adjusted HR, 1.88, 95% CI 0.90–3.91, *P* for interaction = 0.001). This may be related to the high mortality risk in patients with pneumonia, which partially reverses the negative association between the TyG index and mortality. Additionally, when the TyG index was ≥ 8.6 and in the subgroup of patients undergoing surgery, we observed a weaker correlation (adjusted HR, 1.37, 95% CI 1.03–1.82, *P* for interaction < 0.001). This could be attributed to the better baseline health status of patients undergoing surgery, as they had lower proportions of pulmonary infection, sepsis, and shock compared to those not undergoing surgery, resulting in a lower mortality risk. Further research is needed to validate these results in the future.

The mechanism underlying the association between the TyG index and the risk of mortality in critically ill pediatric patients remains incompletely elucidated, and it may be related to the state of IR represented by the TyG index. IR is not a specific response in critically ill patients, and it is involved in various processes such as endothelial dysfunction, oxidative stress, inflammatory responses, coagulation imbalance, and cardiovascular remodeling, all of which can exacerbate the condition of critically ill patients [31, 34, 35]. Hyperglycemia associated with IR is common in patients in ICU [36, 37], and sustained hyperglycemia can promote tissue acidosis, the generation of reactive oxygen and nitrogen species, and infiltration of inflammatory cells, leading to more severe structural and functional tissue damage [38]. Prior research has indicated that intensified insulin therapy may reduce the risk of in-hospital mortality [37].

The strength of this study was the inclusion of pediatric patients across all age groups under 18 years old, excluding neonates, which enhanced the generalizability of the results. Multiple sensitivity and additional analyses further ensured the robustness of the findings. However, several limitations should be acknowledged. Firstly, this study was a retrospective study, and causal relationships between the TyG index and in-hospital mortality and in-ICU mortality cannot be inferred. Secondly, the study only included patients from a pediatric hospital in Zhejiang Province, China. Therefore, the applicability of the results to other countries and regions remains unclear. Future multi-center studies with diverse racial populations are needed to verify the findings. Thirdly, other unadjusted confounding factors (such as the PRISM and PIM scores) may weaken the correlation between the TyG index and mortality. However, E-value analysis results indicated that the study results were relatively robust and less influenced by other factors. Additionally, the lack of specific cause-of-death information in the database limited further exploration of the association between TyG index and different causes of death. Finally, the absence of long-term outpatient follow-up prevented further investigation between the TyG index and long-term prognosis in pediatric patients.

## Conclusion

Our study indicated that the TyG index was an important risk factor of in-hospital all-cause mortality in critically ill pediatrics. The association between the TyG index and 30-day in-hospital and in-ICU all-cause mortality was nonlinear U-shaped, with a cutoff point at the TyG index of 8.6. These findings suggest that the TyG index may be a novel and important factor for the short-term clinical prognosis in pediatric patients. Prospective studies are essential to validate these findings and assess the TyG index’s utility in clinical practice settings.

### Supplementary Information

Additional file1 (DOCX 29 kb)

## Data Availability

The datasets generated and/or analyzed during the current study are available in the Pediatric Intensive Care database [http://pic.nbscn.org].

## Abbreviations

ICU: Intensive care unit
PRISM: Pediatric risk of mortality
PIM: Pediatric index of mortality
TyG index: Triglyceride-glucose index
IR: Insulin resistance
FBG: Fasting blood glucose
TG: Triglyceride
PIC: Pediatric intensive care
ZUCH: Children’s Hospital, Zhejiang University School of Medicine
GICU: General intensive care unit
SICU: Surgical intensive care unit
CICU: Cardiac intensive care unit
NICU: Neonatal intensive care unit
LOS hospital: Length of hospital stay
LOS ICU: Length of intensive care unit stay
ICD-10: International classification of diseases 10
BUN: Blood urea nitrogen
SCr: Serum creatinine
WBC: White blood cell count
hs-CRP: High-sensitivity C-reactive protein
SD: Standard deviation
IQR: Interquartile range
ANOVA: Analysis of variance
HR: Hazard ratio
CI: Confidence interval
RCS: Restricted cubic spline
AKI: Acute kidney injury
KDIGO: Kidney disease improving global outcomes
MIMIC: Medical information mart for intensive care
