# Supplementary material for: U-shaped association between triglyceride-glucose index and all-cause mortality among critically ill pediatrics: a population-based retrospective cohort study
Source: Cardiovasc Diabetol. 2024 Jun 26;23:222. doi: 10.1186/s12933-024-02310-2 (PMC11210025; doi:10.1186/s12933-024-02310-2)
Supplement: Supplementary file 1 — Additional file1 (DOCX 29 kb) [file 12933_2024_2310_MOESM1_ESM.docx]

**Supplementary Online Content**

**Supplementary Table 1**. Study population characteristics stratified by TyG index and 30-day in-hospital all-cause mortality.

**Supplementary Table 2**. Sensitivity analyses of the association between TyG index and 30-day in-hospital all-cause mortality.

**Supplementary Table 1. Study population characteristics stratified by TyG index and 30-day in-hospital all-cause mortality.**

|  | **TyG index <8.6** | | **TyG index** $\boldsymbol{\geq}$**8.6** | |
| --- | --- | --- | --- | --- |
|  | **30-day in-hospital all-cause mortality** | | **30-day in-hospital all-cause mortality** | |
|  | No (N=2672) | Yes (N=108) | No (N=2790) | Yes (N=136) |
| TyG index | 8.1±0.4 | 7.9±0.9 | 9.1±0.5 | 9.40 (0.73) |
| Male, % | 1587 (59.4) | 71 (65.7) | 1527 (54.7) | 84 (61.8) |
| Age, months | 24.4 [4.5, 69.7] | 11.0 [3.2, 43.9] | 17.5 [5.1, 55.8] | 16.8 [4.5, 71.3] |
| **Age category (%)** |  |  |  |  |
| 1 month-1 year | 959 (35.9) | 55 (50.9) | 1172 (42.0) | 53 (39.0) |
| 1 year-5 years | 931 (34.8) | 28 (25.9) | 978 (35.1) | 43 (31.6) |
| $\geq$5 years | 782 (29.3) | 25 (23.1) | 640 (22.9) | 40 (29.4) |
| **ICU types (%)** |  |  |  |  |
| GICU | 573 (21.4) | 54 (50.0) | 662 (23.7) | 68 (50.0) |
| CICU | 329 (12.3) | 4 (3.7) | 548 (19.6) | 2 (1.5) |
| NICU | 229 (8.6) | 4 (3.7) | 99 (3.5) | 3 (2.2) |
| PICU | 731 (27.4) | 35 (32.4) | 630 (22.6) | 54 (39.7) |
| SICU | 810 (30.3) | 11 (10.2) | 851 (30.5) | 9 (6.6) |
| **Laboratory** |  |  |  |  |
| Albumin, g/L | 41.6 [36.8, 45.0] | 37.5 [32.5, 41.0] | 41.8 [37.1, 45.3] | 36.1 [29.2, 39.9] |
| BUN, mmol/L | 3.70 [2.71, 4.83] | 3.94 [3.11, 6.15] | 3.70 [2.50, 5.02] | 4.53 [2.96, 6.24] |
| Cystatin-C, mg/dL | 0.83 [0.68, 1.10] | 0.99 [0.66, 1.44] | 0.92 [0.75, 1.21] | 1.03 [0.79, 1.40] |
| SCr, mmol/L | 44.0 [36.0, 52.0] | 43.3 [30.6, 57.0] | 43.0 [37.0, 52.0] | 46.0 [33.1, 66.8] |
| Total cholesterol, mmol/L | 3.44 [2.75, 4.12] | 2.74 [2.11, 3.61] | 3.79 [3.12, 4.47] | 3.24 [2.62, 4.02] |
| Hemoglobin, g/L | 115.0 [100.0, 127.0] | 110.0 [93.8, 123.0] | 114.0 [99.0, 125.0] | 102.0 [83.8, 118.0] |
| hs-CRP, mg/L | 3.0 [1.0, 10.0] | 5.0 [1.0, 35.3] | 3.0 [1.0, 8.0] | 80 [3.0, 42.3] |
| Lymphocyte, % | 38.6 [21.0, 54.1] | 30.1 [16.5, 41.9] | 46.9 [28.2, 62.0] | 33.6 [18.3, 53.0] |
| Neutrophil, % | 51.6 [34.1, 72.2] | 61.7 [48.1, 78.3] | 41.8 [26.7, 62.3] | 57.5 [34.1, 75.3] |
| Platelet, 10^9^/L | 308.0 [231.0, 386.0] | 265.0 [135.0, 355.3] | 322.0 [232.0, 413.0] | 226.5 [72.0, 353.0] |
| WBC,10^9^/L | 8.8 [6.6, 12.0] | 10.6 [6.6, 15.9] | 9.2 [6.9, 12.3] | 9.5 [5.6, 15.9] |
| **Surgical procedures (%)** |  |  |  |  |
| Cardiac | 219 (8.2) | 2 (1.9) | 402 (14.4) | 1 (0.7) |
| Gastrointestinal | 122 (4.6) | 0 (0.0) | 92 (3.3) | 0 (0.0) |
| Neurosurgical | 247 (9.2) | 5 (4.6) | 267 (9.6) | 2 (1.5) |
| Respiratory | 48 (1.8) | 0 (0.0) | 50 (1.8) | 1 (0.7) |
| Other | 46 (1.7) | 0 (0.0) | 79 (2.8) | 0 (0.0) |
| **Comorbidities (%)** |  |  |  |  |
| Congenital valvular heart disease | 24 (0.9) | 0 (0.0) | 43 (1.5) | 0 (0.0) |
| Congenital heart disease | 279 (10.4) | 9 (8.3) | 469 (16.8) | 6 (4.4) |
| Pneumonia | 296 (11.1) | 33 (30.6) | 274 (9.8) | 25 (18.4) |
| Malignant tumors | 75 (2.8) | 5 (4.6) | 188 (6.7) | 15 (11.0) |
| Sepsis | 77 (2.9) | 3 (2.8) | 89 (3.2) | 8 (5.9) |
| Shock | 14 (0.5) | 0 (0.0) | 14 (0.5) | 1 (0.7) |
| Vasopressors use, % | 448 (16.8) | 31 (28.7) | 590 (21.1) | 34 (25.0) |
| LOS hospital, days | 11.0 [7.0, 18.0] | 8.0 [4.0, 16.3] | 11.0 [7.0, 18.0] | 9.5 [4.8, 18.0] |
| LOS ICU, days | 2.8 [0.9, 7.9] | 7.9 [4.0, 16.0] | 2.0 [0.9, 6.7] | 7.9 [4.0, 17.2] |
| Days from hospital admission to ICU admission | 1.0 [0.0, 3.0] | 0.0 [0.0, 0.0] | 2.0 [0.0, 3.0] | 0.0 [0.0, 0.0] |

**Abbreviations:** TyG, triglyceride-glucose; ICU, intensive care unit; GICU, general intensive care unit; PICU, pediatric intensive care unit; SICU, surgical intensive care unit; CICU, cardiac intensive care unit; NICU, neonatal intensive care unit; BUN, blood urea nitrogen; SCr, serum creatine; WBC, white blood cell count; hs-CRP, high sensitivity C reactive protein; LOS, length of stay.

**Supplementary Table 2. Sensitivity analyses of the association between TyG index and 30-day in-hospital all-cause mortality.**

| **TyG index**  **(per unit increase)** | **Total, N** | **No. of events (incident rate, %)** | **Crude model** | | **Model 1** | | **Model 2** | |
| --- | --- | --- | --- | --- | --- | --- | --- | --- |
|  |  |  | **HR [95% CI]** | **P value** | **HR [95% CI]** | **P value** | **HR [95% CI]** | **P value** |
| **Sensitivity analysis 1: Excluding individuals at high risk of death^*^ (N=4636)** | | | | | | | | |
| <8.6 | 2295 | 67 (2.9) | 0.61 [0.49, 0.74] | <0.001 | 0.60 [0.47, 0.77] | <0.001 | 0.61 [0.47, 0.78] | 0.001 |
| $\geq$8.6 | 2341 | 87 (3.7) | 2.38 [1.83, 3.11] | <0.001 | 1.99 [1.50, 2.65] | <0.001 | 1.69 [1.24, 2.30] | <0.001 |
| **Sensitivity analysis 2: Excluding individuals at high risk of death or undergo AKI (N=4255)** | | | | | | | | |
| <8.6 | 2092 | 49 (2.3) | 0.60 [0.48, 0.76] | <0.001 | 0.59 [0.45, 0.79] | <0.001 | 0.60 [0.45, 0.80] | 0.001 |
| $\geq$8.6 | 2163 | 62 (2.9) | 2.57 [1.90, 3.49] | <0.001 | 2.13 [1.53, 2.97] | <0.001 | 1.84 [1.29, 2.63] | <0.001 |
| **Sensitivity analysis 3: Excluding individuals with LOS hospital ≤3 days (N=5459)** | | | | | | | | |
| <8.6 | 2652 | 95 (3.6) | 0.71 [0.56, 0.92] | 0.009 | 0.74 [0.56, 0.99] | 0.041 | 0.73 [0.55, 0.98] | 0.037 |
| $\geq$8.6 | 2807 | 114 (4.1) | 1.87 [1.43, 2.45] | <0.001 | 1.70 [1.29, 2.24] | <0.001 | 1.45 [1.08, 1.95] | 0.013 |
| **Sensitivity analysis 4: Using dataset before imputation. (N=5570)** | | | | | | | | |
| <8.6 | 2715 | 105 (3.9) | 0.68 [0.55, 0.84] | <0.001 | 0.72 [0.56, 0.93] | 0.012 | 0.72 [0.55, 0.93] | 0.013 |
| $\geq$8.6 | 2855 | 128 (4.5) | 2.03 [1.59, 2.60] | <0.001 | 1.77 [1.37, 2.29] | <0.001 | 1.47 [1.12, 1.93] | 0.006 |

^*^Individuals at high risk of death was defined as those with malignant tumors, sepsis, shock, or pneumonia.

AKI was defined as serum creatinine during the follow-up ≥1.5 fold baseline serum creatinine or need for dialysis according to the Kidney Disease: Improving Global Outcomes (KDIGO) clinical guidelines.

Model 1 adjusted for age, sex, ICU types, surgical procedure, congenital valvular heart disease, congenital heart disease, malignant tumors, pneumonia, sepsis, shock, use of vasopressors, white blood cell count, lymphocyte percentage, neutrophil percentage, and high sensitivity C reactive protein.

Model 2 further adjusted for serum albumin, total cholesterol, hemoglobin, platelets, blood urea nitrogen, cystatin C, and serum creatinine.
